# Knowledge, Practice, and Associated Factors of Essential Newborn Care among Sudanese Women in Eastern Sudan

**DOI:** 10.3390/children9060873

**Published:** 2022-06-12

**Authors:** Abdullah Al-Nafeesah, Mohammed Ahmed A. Ahmed, Omer Elhory, Hyder M. Mahgoub, Bahaeldin A. Hassan, Osama Al-Wutayd, Ishag Adam

**Affiliations:** 1 Department of Pediatrics, Unaizah College of Medicine and Medical Sciences, Qassim University, Unaizah 56219, Saudi Arabia; 2 Faculty of Medicine, Gadarif University, Gadarif 425, Sudan; wadaeis2@gmail.com (M.A.A.A.); elhory73@hotmail.com (O.E.); hdrmahgoub@yahoo.com (H.M.M.); 3 Department of Obstetrics and Gynecology, College of Medicine, King Khalid University, Abha 62529, Saudi Arabia; bahasuikt@hotmail.com; 4 Department of Family and Community Medicine, Unaizah College of Medicine and Medical Sciences, Qassim University, Unaizah 56219, Saudi Arabia; o.alwutayd@qu.edu.sa; 5 Department of Obstetrics and Gynecology, Unaizah College of Medicine and Medical Sciences, Qassim University, Unaizah 56219, Saudi Arabia; ishagadam@hotmail.com

**Keywords:** breastfeeding, cord care, knowledge, newborn care, practice, thermal care

## Abstract

(1) Background: There is a high neonatal mortality rate in countries with low resources, especially sub-Saharan countries. There is no published data in Sudan on mothers’ knowledge and practice of essential newborn care. This study aimed to assess the maternal knowledge and practice of essential newborn care in Gadarif city, eastern Sudan. (2) Methods: A cross-sectional study was conducted in Gadarif city, eastern Sudan. Postnatal mothers (384) were recruited from postnatal and vaccination clinics. A structured questionnaire was used to collect the data. Mothers who responded to essential newborn care knowledge and practice items at a rate equal to 75% or above were classified as having good knowledge and practice. Logistic regression analysis was performed to identify the factors associated with essential newborn care knowledge and practice. (3) Results: In this study, 268 (66.4%) and 245 (63.8%) of the 384 participants had good knowledge and practice of essential newborn care, respectively. None of the investigated factors (age, residence, education, occupation, parity, antenatal care, and mode of delivery) was associated with knowledge and practice of essential newborn care with sociodemographic and obstetric factors. Mothers with poor knowledge were less likely to have good practices (adjusted odds ratios = 0.41; 95% CI (0.26–0.64)). The reported malpractices were giving dietary supplements to the babies (48.2%), mainly water (40.0%) and cow’s milk (43.2%), and putting substances on the umbilical cord (62.8%), with butter (92.1%) accounting for the majority. (4) Conclusion: In the present study, around two-thirds of the participants had good essential newborn care knowledge and practice. Poor knowledge was less likely to be associated with good newborn care practices. More research is needed to build baseline data for neonatal mortality reduction plans.

## 1. Background

Every year, 2.6 million newborn deaths occur globally, the majority of which are in low-setting countries [1]. The global under-five mortality rate dropped by 43 per 1000 deaths between 1990 to 2010 [2]. However, African countries still report the highest rate of under-five and neonatal mortality [3]. Essential newborn care (ENC) plays a vital role in reducing neonatal mortality [3]. ENC, as recommended by the World Health Organization (WHO), includes the early initiation of exclusive breastfeeding, hygienic cord care, and thermal care [4]. Thermal care components are the immediate wrapping and drying of the baby, delaying bathing for 24 h, and skin-to-skin contact (SSC) [5]. Breastfeeding and SSC have been proven to be effective, low-cost interventions to save newborn lives [6]. Despite this, SSC practice was reported to be suboptimal universally [7]. In Sudan, the last household survey conducted in 2010 reported 189 neonatal deaths out of 6198 live births [8]. Poor household wealth index and delivery complications are the major risk factors associated with neonatal mortality in Sudan [8]. Different malpractices of ENC were reported in African countries, including bathing newborns immediately after delivery and applying traditional substances to the cord [7]. Educated urban residents and good antenatal care attendants were associated with good ENC practice [9,10]. Training in ENC among healthcare workers is needed in African countries. Lower staff members’ knowledge of newborn care and low resources were observed [11]. In 2019, the WHO recommended going back to the basics for newborn care in Sudan [12].

Several studies addressed ENC in the sub-Saharan [13,14,15,16,17,18] To our knowledge, there is no published study on the knowledge and practice of ENC among Sudanese women. This study aimed to assess the knowledge and practice of ENC among Sudanese women and its related determining factors.

## 2. Methodology

### 2.1. Study Design and Settings

A cross-sectional study was conducted in Gadarif city, eastern Sudan, from November 2020 to January 2021. Gadarif has a population of 1,727,401 residents. The city is located 400 km from the capital, Khartoum, on the Ethiopian border. Data were recruited from the postnatal and vaccination clinics distributed all over the city.

### 2.2. Outcome Measures

The main outcome measures were the knowledge and practice of ENC by the mothers. Secondary outcomes were factors associated with ENC knowledge and practice.

### 2.3. Sample Size

The sample size (384) was calculated using the sample size for cross-sectional studies [19]. The calculation was guided by the expected prevalence of ENC practice (50%) among a population, based on a similar study in the region [20]. This sample had a 95% confidence level and a 5% confidence limit.

### 2.4. Study Population

**Inclusion criteria:** This study included all healthy mothers in the first 6 weeks of their postnatal period who attended the postnatal and vaccination clinics during the study period. Mothers who delivered live babies and were willing to participate in the study were included.

**Exclusion criteria:** Severely ill mothers and mothers who had stillbirths or babies with congenital malformations at the time of the study were excluded.

### 2.5. Data Collection

Questionnaires were used to collect the data. Trained medical officers were allocated to fill out the questionnaire throughout the interview. The first part of the questionnaire was composed of sociodemographic and obstetric data. The second part of the questionnaire was an assessment of each participant’s knowledge and practice of ENC. Each ENC practice was assessed with a series of closed questions, scoring one (1) and zero (0) points for appropriate and inappropriate responses, respectively. The scoring system was generated to obtain knowledge about ENC, with categories of poor or good knowledge. Knowledge was assessed using questions that addressed three components of newborn care, per the WHO recommendations. Good breastfeeding includes the first-hour initiation of exclusive breastfeeding. Optimal thermal care was defined as SSC, using the kangaroo method, and the delay of bathing for 24 h. Safe cord care practice was described as cutting and tying the cord with sterilized materials and to refrain from applying substances on it. The score was estimated as “1 = Correct response (following WHO guidelines) and 0 = Incorrect response (not included in WHO guidelines)”. Participants with at least 75% correct responses were considered as having good knowledge, while those with fewer than 75% correct responses were considered mothers with poor knowledge.

Good practice was assessed through the three components of ENC (cord care, optimal thermal care, and exclusive breastfeeding), based on WHO recommendations [4]. Mothers were categorized as good practitioners if they achieved 75% of the appropriate responses to the questionnaire items. These items were consistent with WHO recommendations for good ENC practice. Poor practice was a category of mothers with fewer than 75% appropriate responses. This scoring was guided by similar studies [20,21].

### 2.6. Statistics

The data were entered into a computer, with SPSS version 22 used for analysis. Continuous data were not normally distributed and were expressed as median (interquartile), while the categorized data were expressed as numbers (%). Multivariable logistic regression was performed using practice and knowledge of ENC as the dependent variable and sociodemographic and obstetric factors as the independent variables. Multivariable analysis using a standard logistic regression technique to evaluate the independent effect of each covariate by controlling for the effect of other variables. The adjusted odds ratio (AOR) and 95% confidence intervals were computed. A *p*-value of less than 0.05 in the multivariable logistic regression analysis was considered statistically significant.

### 2.7. Ethical Approval

Ethical approval was obtained from the Ethical Committee of the Faculty of Medicine of Gadarif University, Sudan. Written informed consent was signed by each participant after an explanation of the study’s objectives. The right to withdraw at any stage of the study was ensured.

## 3. Results

### 3.1. Sociodemographic Characteristics

Three hundred eighty-four mothers were enrolled in the study. The median (interquartile) age of the participants was 27.0 (22.0–30.0) years. Over half of the mothers, 256 (66.7%), had less than a secondary education. One hundred forty-four participants (37.5%) were employed, and more than half (57.8%) of their residences were urban (Table 1).

### 3.2. Obstetric Data

Most participants, 326 (84.9%), had antenatal care during the index pregnancy, but the maximum number of antenatal care visits were two in 98.5% of cases. Two hundred and forty-five women (63.8%) were delivered vaginally, with nearly one-third (32.2%) delivered at home (Table 1).

### 3.3. Knowledge of ENC

Breastfeeding: Two hundred sixty-eight (69.8%) participants knew to initiate breastfeeding immediately after delivery. The women had received information about colostrum (90.1%), and 343 (89.3%) were informed about its advantages. Cord care: Three hundred six participants (79.7%) were informed about the material that should be used to cut the umbilical cord. Thermal care: Most mothers (63.8%) believed that keeping the baby in SSC was essential to protect the baby from hypothermia. Information about drying and wrapping babies was reported by 71.1% of participants. However, only 8.9% attained knowledge about delaying bathing the baby for 24 h (Table 2). Overall, 225 (66.4%) participants had good knowledge about ENC, based on their responses to the questionnaire items (Table 2).

### 3.4. Factors Associated with Participants’ Knowledge of ENC

Age, residence, education, occupation, parity, antenatal care, and mode of delivery were not significantly associated with ENC knowledge (Table 3).

### 3.5. ENC Practices among the Study Populations

Breastfeeding: First postnatal hour breastfeeding was practiced by 344 (89.6%) mothers. Half of the mothers (51.8%) adopted exclusive breastfeeding. The majority (93.8%) of participants fed their babies with colostrum. Cord care: The cord was cut with a new razor blade (65.6%) and tied with a clamp or clean thread (79.7%); no materials were applied to it (37.2%). Thermal care: SSC was adopted by 339 (88.3%) postnatal mothers. Nearly half of the mothers (49.0%) wrapped their babies with preprepared towels. Waiting 24 h to bathe the baby was practiced by 66.1% of participants (Table 2). The participants who scored ≥ 75% on the practice items (good practice) were 245 (63.8%) (Table 2).

### 3.6. Factors Associated with Participants’ Practices of ENC

There were no statistically significant associations between age, residence, education, occupation, parity, antenatal care, and mode of delivery with newborn practice. Poor knowledge (AOR = 0.41; 95% CI (0.26–0.64)) was less likely to report good newborn practices (Table 4).

### 3.7. Misconceptions and Malpractices

Only two out of 384 mothers (0.5%) were reluctant to give the first milk to their babies. Beliefs that the first milk could cause diarrhea and decreased growth were behind this reluctance. To cut the cord, 191 (57.7%), 66 (19.9%), and 59 (17.8%) reported the usage of a boiled new razor blade, an un-boiled used razor blade, and available sharp material, respectively. Two hundred forty-one (62.8%) participants applied substances to the umbilical cord. Butter accounted for 92.1% of the applied materials. We observed that 185 (48.2%) mothers fed their babies prelacteals. The main two substances used as an additional diet were water (40.0%) and cow milk (43.2%). The rest of the participants used butter (10.8%) and holly water (6.0%) (Table 5).

## 4. Discussion

The current study indicated that 66.4% of the participants had good ENC knowledge. This finding is comparable to a similar study in Ghana, in which 62% of mothers had good knowledge about ENC [20]. However, the knowledge about ENC in our study was significantly higher than the reported results from Mekelle City, Ethiopia (36.1%) [21].

These variations can be attributed to differences in the study settings and education levels. The demographics in the Ethiopian study showed only 18.2% above secondary education, in comparison with 32.3% in our study population.

Breastfeeding knowledge among the study populations was satisfactory. Most mothers knew about early breastfeeding (69.8%) and colostrum (90.1%) and its advantages (89.3%). Comparable findings were noted in South Sudan [22].

Overall, cord care knowledge was encouraging in the present study. Most mothers (79.7%) knew which was the right instrument used to cut the cord.

Nearly two-thirds of participants had gained good knowledge about thermal care. Similar thermal care knowledge was reported in Ethiopia [21]. Poor knowledge about the newborn’s first bath was reported (8.9%). This compares with common beliefs documented in Nigeria [23].

Information about ENC given to postnatal mothers in our community was mostly delivered by midwives. A previously published study in Sudan reported poor ENC knowledge (50.6%) among midwives [24]. Wrong messages about ENC were expected to be delivered from midwives to postnatal mothers in the present study.

In the present study, good ENC practice was 63.8% overall. This is consistent with findings from a study in Nepal (66.2%) [25]. The prevalence of good ENC practices in our data is higher than in similar studies from Ethiopia, Damot Pulasa (24%) [26], and Aksum Town (26.7%) [16]. These differences could be explained by sociodemographic differences between the study areas, even within the same country.

The current study showed that first postnatal hour breastfeeding was practiced by 344 (89.6%). In agreement with this frequency, previous studies approved the benefit of the early initiation of breastfeeding [6,7,27].

Our data reported that only 51.8% of mothers exclusively breastfed their babies. This is much lower than reported findings from Ghana (87%) [20] and Uganda (93.2%) [28]. Our finding was comparable with results from Egypt (56%) [29] and Ethiopia (50.2%) [26]. The widespread practice of prelacteal feeding in these two neighboring countries was due to wrong beliefs. In Egypt, supplementary feeding was wrongly considered part of exclusive breastfeeding [29].

Colostrum was given by 93.8% in the present study. Similar good practices were observed in Ghana (91.5%) [20] and Uganda (94.7%) [30]. The WHO raised the importance of first milk to the baby [31]. A previous study in Ethiopia revealed that mothers discarded colostrum, as they had negative beliefs about it [26].

Good cord care practices among the study population were documented, including cutting the cord with a new razor blade (65.6%) and tying with a clamp or clean thread (79.7%). Consistent good cord care practices were reported in a Nigerian study [32]. Nearly two-thirds (67.8%) of vaginal deliveries in our study were hospital deliveries. Hospital cord care practice is expected to be better than that of home delivery. A previously published study in Sudan assessed the nurse midwife’s practice of cord care in four major hospitals in Khartoum; good cord care practice was 65.8% [24].

In 2019, the WHO conducted a workshop in Khartoum that recommended the practice of SSC as an effective, non-costly, and highly beneficial form of thermal care [12]. A worldwide study observed the high prevalence of SSC among high- and middle-income countries [33]. The present study reported one of the highest prevalences of SSC practices in Africa (88.3%). The majority, if not all, of studies published in Africa, reported lower prevalences of SSC: Uganda (17.6%) [30], Ethiopia (19%) [34], and Gambia(35.7%) [35]. This relatively high SSC in our data might be related to another finding: early breastfeeding (89.6%). SSC was ensured during early breastfeeding sessions.

The current study indicated that 66.1% of mothers bathe their babies 24 h after birth. Similar practices were demonstrated in Ghana [20] and Ethiopia [10]. However, lower prevalences were observed in Malawi [36] and Nigeria [23]. In countries where the practice of early bathing was adopted, dirty babies and bad odors were the most frequent factors [23].

ENC knowledge was the only association with ENC practice in our study. Poor knowledge (AOR = 0.41; 95% CI (0.26–0.64)) was less likely to report good newborn practice. This finding supports published studies from Ethiopia [10,21] and Nepal [25]. This could be explained by the fact that in the absence of ENC knowledge, malpractices like prelacteal feeding and applying substances to the umbilical cord will dominate. A previously published study in Bossaso, Somalia, noticed the positive effect of maternal ENC knowledge on practice [25].

The malpractice of applying substances to the cord is widely distributed in African communities. Our data reported that 62.8% of participants applied substances to the cord. We found butter (92.1%) at the top of the list of applied substances. The malpractice of applying butter was similarly observed in studies from Ghana [27] and Ethiopia [34]. Other substances, such as powder in South Sudan [22] and Uganda [28], were applied to the umbilical cord. The beliefs behind the application of these substances were enhancing the healing of the cord and accelerating the cord separation process [37].

We observed that 185 (48.2%) of mothers fed their babies prelacteals. Water (40.0%), cow milk (43.2%), butter (10.8%), and holly water (6.0%) were the substances used. These substances varied from one country to another, based on the cultures of the community. A tea and glucose solution was used by mothers in Uganda [28], anise and caraway in Egypt [29], and honey in Ethiopia [10]. The majority of mothers claimed that family and community pressure were the reason behind this malpractice [38].

Interventions to improve the knowledge and practice of ENC in Sudan are needed. An interventional study in Sudan examined the practice of drying and wrapping the baby. Midwives demonstrated a tenfold improvement in the practice, with a subsequent reduction in neonatal mortality [39]. Our study was facility-based, so our results might not reflect community practices. Moreover, the sample size was small, and the single-city design limited generalizability.

## 5. Conclusions

Approximately two-thirds of participants had good ENC knowledge and practice. Although the prevalence of good SSC practices was higher in comparison with other African countries, our study shows insufficient ENC knowledge and practices among Sudanese women.

## Figures and Tables

**Table 1:** Sociodemographic characteristics of the study population.

Variables		Frequency	Percent
Age group, years *	27.0 (22.0–30.0)		
Education			
	≥secondary level	256	66.7
	<secondary level	128	33.3
Occupation			
	Housewives	240	62.5
	Employed	144	37.5
Residence			
	Rural	162	42.2
	Urban	222	57.8
Parity *	2 (1–4)		
Antenatal care when pregnant			
	≥two visits	324	84.4
	˂two visits	60	15.6
Mode of delivery			
	Vaginal delivery	245	63.8
	Cesarean delivery	139	36.2
Place of birth of vaginal delivery			
	Home	79	32.2
	Hospital	166	67.8
Postnatal care			
	Yes	374	97.4
	No	10	2.6
Time of first postnatal care			
	Day 1	86	23.0
	Day 2–3	70	18.7
	Day 7–14	96	25.7
	Day 40–42	122	32.6

* Median (interquartile).

**Table 2:** The quality of ENC components of knowledge and practice.

Components of ENC	Number	Percentage
Knowledge		
Breastfeeding		
Breastfeeding a newborn immediately after delivery	268	69.8
Informed about feeding the first milk	346	90.1
Informed about advantages of first milk	343	89.3
Cord care		
Informed about cutting the cord with new razor blade	306	79.7
Thermal care		
Deliver baby onto mother’s abdomen or into her arms	246	64.1
Dry and wrap the baby	273	71.1
Place the baby in skin-to-skin contact	245	63.8
Delay bathing the baby for 24 h after delivery	34	8.9
Overall good newborn care	255	66.4
Practice		
Breastfeeding		
Breastfeeding started in the first hour of delivery	344	89.6
Exclusive breastfeeding	199	51.8
Colostrum was given to the baby	360	93.8
Newborn was breastfed > 8 times per day	213	55.5
Cord care		
The cord was cut with new razor blade	252	65.6
The cord was tied with clamp/clean thread	306	79.7
No material has been applied to the cord	143	37.2
Thermal care		
The baby positioned on mother’s abdomen	83	21.6
The baby kept in immediate skin-to-skin contact	339	88.3
The baby was wrapped before the delivery of the placenta	137	35.7
The baby was wrapped with pre-prepared towel	188	49.0
The baby had been bathed after 24 h	254	66.1
Overall good newborn care practice	**245**	**63.8**

**Table 3:** Factors associated with participants’ knowledge of ENC.

Variables	Poor Knowledge (129)	Good Knowledge (255)	OR (95%CI)	*p*
Age, year *	27 (21–30)	27 (23–31)	1.02 (0.96–1.08)	0.542
Residence				
Rural	61 (47.3)	101 (39.6)	0.60 (0.34–1.04)	
Urban	68 (52.7)	154 (60.4)	Reference	
Educational level				
≥secondary level	87 (67.4)	169 (66.3)	Reference	
<secondary level	42 (32.6)	86 (33.7)	1.11 (0.62–2.01)	0.712
Occupation				
Housewives	78 (60.5)	162 (63.5)	Reference	
Employee	51 (39.5)	93 (36.5)	0.75 (0.44–1.27)	0.296
Parity *	2.0 (1.0–3.0)	3.0 (1.0–4.0)	1.04 (0.86–1.27)	0.644
Antenatal care				
≥two visits	113 (87.6)	211 (82.7)	Reference	
˂two visits	16 (12.4)	44 (17.3)	1.58 (0.82–3.06)	0.168
Mode of delivery				
Vaginal	87 (67.4)	158 (62.0)	Reference	
Cesarian	42 (32.6)	97 (38.0)	1.29 (0.81–2.04)	0.249

* Median (interquartile).

**Table 4:** Factors associated with participants’ practices of ENC.

Variables	Poor Practice 139	Good Practice 245	OR (95%CI)	*p*
Age, year *	27 (21–30)	27 (23–31)	0.96 (0.91–1.01)	0.144
Residence				
Rural	63 (45.3)	99 (40.4)	0.75 (0.43–1.30)	
Urban	76 (54.7)	146 (59.6)	Reference	
Educational level				
≥secondary level	92 (66.2)	164 (66.9)	Reference	
<secondary level	47 (33.8)	81 (33.1)	0.93 (0.51–1.68)	0.815
Occupation				
Housewives	85 (61.2)	155 (63.3)	Reference	
Employee	54 (38.8)	90 (36.7)	0.94 (0.55–1.60)	0.833
Parity *	2 (1–3)	3.0 (1–4)	1.04 (0.86–1.26)	0.652
Antenatal care				
≥two visits	116 (83.5)	208 (84.9)	Reference	
˂two visits	23 (16.5)	37 (15.1)	0.83 (0.45–1.54)	0.567
Mode of delivery				
Vaginal	92 (66.2)	153 (62.4)	Reference	
Cesarian	47 (33.8)	92 (37.6)	1.09 (0.69–1.72)	0.685
Knowledge				
Good	75 (54.0)	180 (73.5)	Reference	
Poor	64 (46.0)	65 (26.5)	0.41 (0.26–0.64)	0.005

* Median (interquartile).

**Table 5:** Misconceptions and malpractices of Sudanese women towards ENC.

Variables	Frequency/Total Events	Percentage
Reasons for not using first milk		
Causes diarrhea	1/2	50
Causes constipation	-	-
Decreases growth	1/2	50
Others	-	-
Non-recommended material used to tie the cord		
Cord tie	21/78	26.9
Others	57/78	73.1
Materials applied to the cord after cutting		
Cow dung	12/241	5.0
Dust	4/241	1.7
Butter	222/241	92.1
Others	3/241	1.2
Additional diet (prelacteal) within 28 days		
Water	74/185	40.0%
Butter	20/185	10.8%
Cow milk/commercial milk formula	80/185	43.2%
Holly water	11/185	6.0%

## Data Availability

The data presented in this study are available on request from the corresponding author.
